# Modeling Debris Flow Events in the Rio Inferno Watershed (Italy) Through UAV-Based Geomorphological Survey and Rainfall Data Analysis

**DOI:** 10.3390/s25071980

**Published:** 2025-03-22

**Authors:** Laura Turbessi, Battista Taboni, Gessica Umili, Giandomenico Fubelli, Anna Maria Ferrero

**Affiliations:** Department of Earth Sciences, University of Turin, Via Valperga Caluso 35, 10125 Turin, Italy; battista.taboni@unito.it (B.T.); gessica.umili@unito.it (G.U.); giandomenico.fubelli@unito.it (G.F.); anna.ferrero@unito.it (A.M.F.)

**Keywords:** debris flow, Rio Inferno, Mt. Chaberton, photogrammetric survey, HEC-RAS

## Abstract

This paper presents an analysis of the debris flow phenomena in the Rio Inferno watershed (Municipality of Cesana Torinese, Western Alps, Italy). The annual frequency and magnitude of these events have caused significant damage to the viability of the historic Chaberton Military Road, which is now closed to transit. This study delved into the processes governing debris flows in the Rio Inferno watershed through detailed geomorphological analysis, an unmanned aerial vehicle (UAV) photogrammetric survey, and the elaboration of rainfall data from the nearby weather monitoring stations. The Hydrologic Engineering Center’s River Analysis System (HEC-RAS) code was used to simulate debris flow events considering critical precipitations associated with return periods of 20, 50, 100, and 200 years, based on the highly detailed topographical model obtained by means of photogrammetry. The paper highlights the importance of studying debris flow phenomena to implement effective risk mitigation and management strategies, especially in the context of climate change and the increased vulnerability of mountain territories.

## 1. Introduction

In recent years, unmanned aerial vehicles (UAVs) have become indispensable in geosciences, offering high-resolution, cost-effective, and rapid data acquisition. Their applications span multiple disciplines, including earthquake studies, where they enable rapid assessment of surface ruptures and fault displacements [1], and flood monitoring, where they aid in mapping inundated areas and analyzing water dynamics [2]. UAVs are also crucial for monitoring rockfalls and landslides, providing detailed displacement measurements and risk evaluations [3,4]. In volcanology, they facilitate the safe collection of thermal imagery, gas emissions, and topographic changes, supporting hazard assessments [5]. Additionally, UAVs enhance geomorphological studies by enabling detailed analysis of landforms [6]. Their ability to operate in remote and hazardous environments makes them a transformative tool for geoscientific research, improving risk assessment and landscape monitoring.

The increase in global temperatures over recent decades has triggered an exponential rise in extreme natural events, such as intense and prolonged rainfalls and the rapid retreat of glaciers [7,8,9]. These phenomena have destabilized and continue to destabilize mountain slopes, promoting the triggering of rapid gravitational movements, such as landslides and debris flows. These events have significant impacts on the morphological evolution of mountainous areas, often urbanized, thus exposing them to a high risk of potentially dangerous landslide events [10].

Debris flows are a typical phenomenon of mountainous areas and are characterized by high velocity [11,12,13]. Their erosive capacity constantly increases their volume and the potentially involved area [14,15]. The impulsive nature of these phenomena generates a series of flows that overlap with each other, producing characteristic deposits with multiple edges and ridges. In the Western Alps, these events are generally triggered by intense rainfalls generating water flows that transport any detrital material they encounter during their runoff and erode the bottom of pre-existing channels. These channels, incised by previous events, concentrate the flow and increase its flow rate, promoting erosion and transport of large amounts of detrital material. The thick and steep postglacial detrital deposits [16] typical of high-altitude mountain environments, together with intense summer precipitation [17], create ideal conditions for the triggering and development of these phenomena. Due to their unpredictability, extremely rapid mobilization, and the large volume of material involved, debris flows represent one of the most devastating landslide processes on Earth. In urbanized areas, debris flows cause enormous economic damage and loss of human lives [18]. According to the Italian High Institute for Environmental Protection and Research (ISPRA) statistics of 2021 [19], in the Piedmont region, 11.1% of all recorded landslide events are debris flows. Based on data from Population at Risk from Landslides and Floods in Italy (POLARIS) [20], numerous events between 1974 and 2023 have caused the evacuation of more than 250 people and the loss of at least 5 human lives.

The Rio Inferno watershed, located uphill of Fenils (Municipality of Cesana Torinese, Western Alps, Italy), is a typical example of an area annually affected by violent debris flows triggered by summer storm events [21]. These flows move downstream, often reaching the confluence with the Dora di Cesana River, located very close to the settlement. In the last 6 years, more than 300 landslide events have affected the municipality of Cesana Torinese, involving about 48 families and more than 230 buildings (ISPRA data) [19]. Among these phenomena, approximately 8% are debris flow events [22].

In general, debris flow risk mitigation strategies are based on the analysis of the geological and geomorphological features of the watershed and the study of precipitation and surface water runoff within it [23,24,25,26].

This research delves into the dynamics of debris flows in the Rio Inferno watershed through field surveys, modeling, and the development of a detailed geomorphological map. This study characterizes both active and inactive processes on the site and identifies the key agents responsible for landscape shaping. Water discharges have been quantified through the analysis of critical rainfall events that have occurred in the watershed over the past 200 years. The application of the software HEC-RAS 6.5 (U.S. Army Corps of Engineers) [27] enabled the creation of models that simulate debris flow events in the area, defining their characteristic parameters and maximum achievable extents.

## 2. Case Study

**Figure 1:**
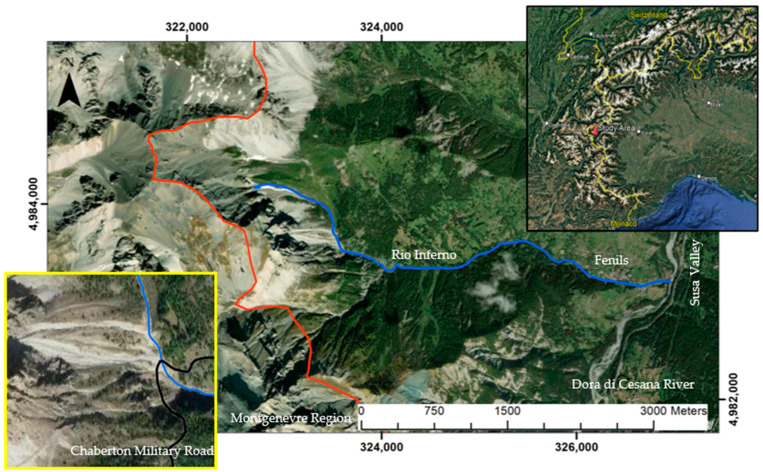
Satellite image showing the Rio Inferno valley in relation to the urbanized area of Fenils and the Italian–French border (in red). The yellow square is a zoomed-in view of the studied area, highlighting the intersection between the Chaberton Military Road (in black) and the Rio Inferno (indicated by a blue line). The black square on the right is a Google Earth satellite image showing the study area in relation to the Piedmont Region and the neighboring national borders. Coordinates in WGS 84 UTM zone 32 N.

The studied area is located in western Piedmont (Italy), in the Fenils’ hamlet, part of the municipality of Cesana Torinese in the upper Susa Valley and close to the French border. Fenils is situated on the bottom of a secondary valley descending from Mt. Chaberton (Figure 1).

The analyzed area extends for approximately 1.5 km^2^ at about 2000 m above sea level. It is crossed by a wide trail, the Chaberton Military Road, which connects the Fenils settlement to the summit of Mt. Chaberton, located in Montgenèvre, a neighboring French municipality. The area is crossed by a primary watercourse, the Rio Enfer or Rio dell’Inferno (Rio Inferno), which flows from northwest to east, branching northward into a series of minor mountain streams.

It is reasonable to assume that there are no aquifers present in the area due to its steepness. The waters flow into small first-order streams with a torrential dynamic and end up in the Dora di Cesana River near Fenils, forming a convergent drainage. These watercourses are intermittent, except for the Dora di Cesana River, which is perennial.

Rainfalls in the area are concentrated in summer and autumn. Due to the high altitude, snow remains on-site for most of the year, except for the period from July to October. The daily temperature range is approximately 10 °C. During the summer, maximum temperatures reach 20–25 °C, while in winter, maximum temperatures do not exceed 15 °C [28].

**Figure 2:**
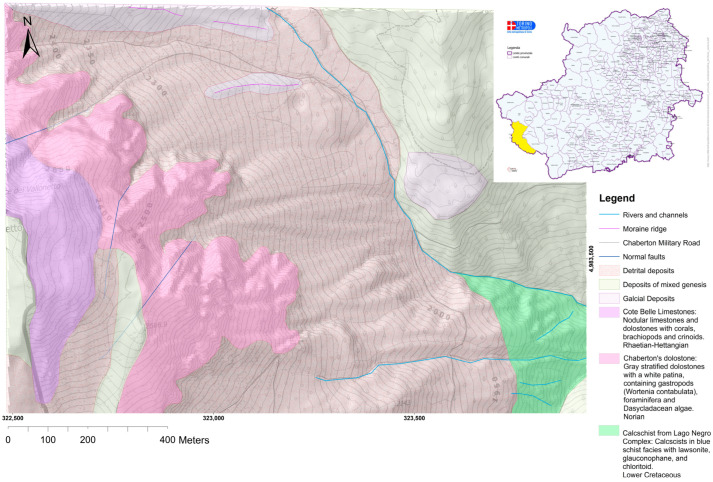
Geological map of the area at a 1:1500 scale, with an explanatory legend of the outcropping geological units and deposits. Coordinates are in WGS 84 UTM zone 32 N.

The studied area includes continental margin geological units, which originate from distal continental margin sectors (European origin) [29]. These units consist of Triassic dolomitic successions, followed by a limestone series ranging in age from the Rhaetian to the Cretaceous. Specifically, the reference unit is known in the literature as the Chaberton-Grand Hoce-Grand Argentier tectonostratigraphic unit [29] and is described in the sheet Cesana 171 of the Carta Geologica d’Italia [30]. This unit outcrops east of Mt. Chaberton and extends to the Montgenèvre region. In this sector of the Western Alps, the steep and fractured dolomitic and carbonate walls release large amounts of detrital material, mostly through rockfalls. This material accumulates at the base of the rock walls (Figure 2). The Chaberton-Grand Hoce and Grand Argentier tectonostratigraphic unit is thrusted over the Albergian Unit via an east-verging thrust and is laterally bounded by high-angle structures with a NE-SW orientation. The evolution of these direct fault systems at the mesoscopic scale represents the responsible phenomenon for the faulting and fracturing of the carbonate walls [21].

From a geomorphological perspective, the area displays various shapes, mostly depositional, which represent the expression of recent and past geomorphological processes.

**Figure 3:**
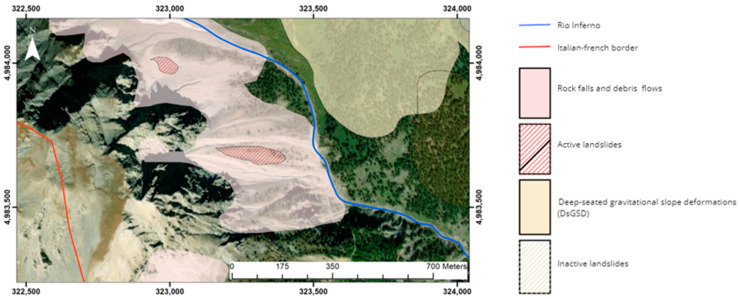
Landslide presence delineated by SIFRAP [31] for the studied area. It can be observed that more than 50% of the area is characterized by gravity-driven deposits, highlighting how the morphogenetic agent represented by gravity governs the geomorphology of the region. Coordinates are in WGS 84 UTM zone 32 N.

As a mountainous zone, the primary morphogenetic agents responsible for these processes are glaciers and gravity. Based on the geomorphological map of the Landslide Information System of Piedmont (SIFRAP) [31], the area under examination is characterized by abundant active debris flow phenomena (Figure 3). During the Pleistocene, the site was covered by the Dora and Chisone glaciers [30]. In addition to gravity and ice, surface waters also played a significant role in shaping the area. Rio Inferno, in particular, generates steep erosion scarps by crossing deposits and the substrate along its path.

**Figure 4:**
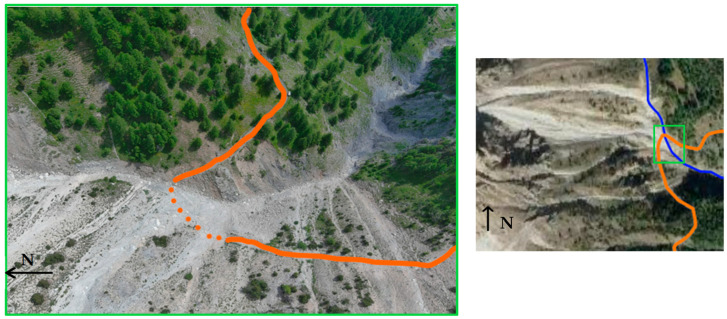
Drone photograph showing the Chaberton Military Road in orange and the ford, completely removed by the debris flow on 1 July 2019, with dashed lines. The panel on the right is located in the same position as the yellow square in Figure 1 and shows, in green, the position of the image on the left within the watershed. The blue line indicates the Rio Inferno.

Fenils’ hamlet has been significantly and multiple times affected in recent decades by debris flows, as evidenced by official documentation from the Consorzio Forestale Alta Val Susa (C.F.AV.S.). One of the most exposed anthropogenic elements is the historic Chaberton Military Road, which suffered the complete removal of the Rio Inferno ford in the summer of 2019 (1 July 2019) as the result of the development of an extensive debris flow (Figure 4). The road has been closed to traffic since then.

## 3. Materials and Methods

**Figure 5:**
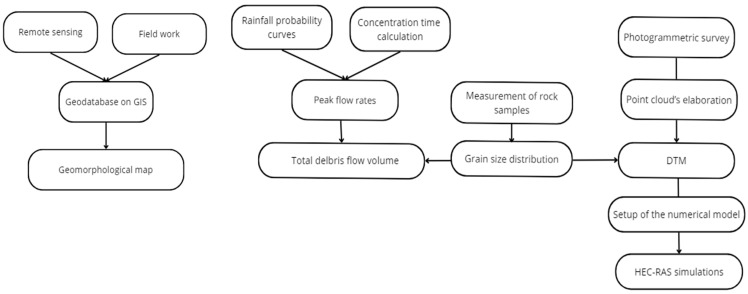
Schematic representation of the workflow, divided into the production of the geomorphological map and the execution of simulations in the HEC-RAS environment.

Since there are no specific data in the reports related to past events, a back analysis of the debris flows that annually affect the study area can help to identify the main quantitative characteristics of the phenomena. To realize the back analysis, a photogrammetric survey was first conducted to obtain detailed DTM for the Rio Inferno watershed. This was necessary for carrying out a remote sensing study aimed at creating a specific geomorphological map for the studied area. Additionally, it allowed the development of HEC-RAS models to define the maximum extent of debris flows and quantify their characteristic parameters. A schematic diagram of the workflow is shown in Figure 5.

### 3.1. Geomorphological Map of the Rio Inferno Watershed

A detailed geomorphological map of the Rio Inferno watershed was first produced, with the dual purpose of identifying the morphogenetic agents acting in the area and their morphological products and obtaining a reference cartography for the numerical modelling of the flow. The geomorphological analysis was carried out by combining pieces of information from the geological literature of the Western Alps, field observations, and morphological data. These were obtained by processing the regional 5 m grid Digital Terrain Model (DTM), orthophotos, and topography provided by the Piedmont Regional Geoportal [32]. In particular, from the DTM, the hillshade and slope maps were produced through a Geographic Information System (GIS) software and later used as the basis for remote shape recognition. The symbology employed in the final geomorphological map was taken from the guidelines proposed by Campobasso et al. [33]. After the creation of a preliminary paper map in the field, a digital equivalent was developed at a 1:1500 scale using ArcMap 10.8.2 (Esri) software, supported by additional graphic programs such as QGIS 3.22.11 [34] and AutoCAD 2021 (Autodesk).

To generate the polygonal, linear, and point geomorphological feature classes, a dedicated geodatabase was created, containing three main domains:

- Observed landforms → classified into erosional and depositional forms;
- Morphogenetic agents → categorized as gravity, surface water, ice, snow, and anthropogenic;
- State of activity → active or inactive.

The resulting map differentiates the landforms observed based on the morphogenetic agent that created them and their state of activity.

The cartographic work produced in this specific case is useful both for obtaining an overall picture of the area and for creating HEC-RAS models that simulate the evolution of debris flows affecting the Chaberton Military Road near the Rio Inferno’s ford. The geomorphological map is the foundation for studies that will provide useful results for designing risk mitigation strategies that may allow the safe road reopening.

### 3.2. Flow Volume and Maximum Flow Rate

To quantify the volume of the flow processes featured in the study area, the following method was employed.

**Figure 6:**
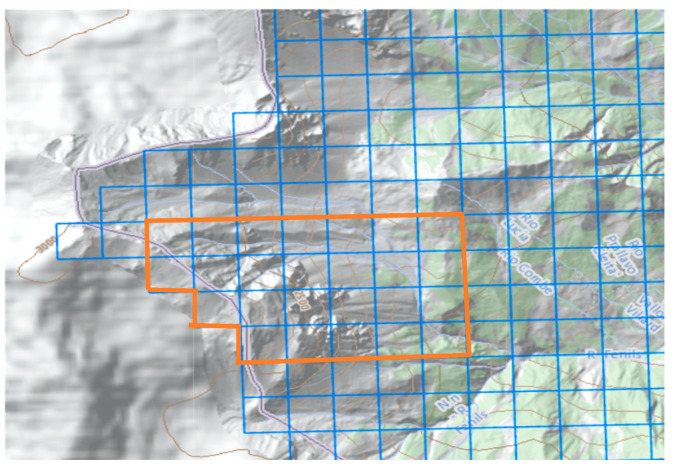
The selected cells (in orange) for the rainfall data extraction on the regional portal [28]. Total extension is 1.56 km^2^.

All the available data consulted [31] pertains to events that reached the built-up area of Fenils and the confluence with the Dora River. However, since the study area is located upstream, these data correspond to a location much further downstream and are therefore not relevant to this study. In the absence of reliable volumes previously calculated, pre-post event DTMs, and without the possibility of obtaining estimates through other means, the analysis started by examining precipitations in the watershed considered. To achieve this, data collected from the regional pluviometric network instruments were used [28]. The portal collects data from climatic stations across the region, providing processed data divided into 205 × 205 m grid cells, covering the entire regional territory. For this study, rainfall data from 25 cells covering the entire area of interest were analyzed. The maximum annual precipitation values for durations of 1, 3, 6, 12, and 24 h were extrapolated from each cell for return periods of 20, 50, 100, and 200 years. Such data, in mm, were averaged to obtain global values reflecting the conditions of the entire study area (Figure 6).

These data were processed using the method proposed by Gumbel [35] to derive the precipitation probability curve for the critical rainfall associated with a certain return period. A precipitation probability curve was obtained for each considered return period; each curve is defined as follows:

*h* = *at^n^* (1)

where *h* is the rainfall height (in mm) and is a function of time (*t*, in *h*) and two adimensional parameters (*a* and *n*). Critical rainfall refers to the precipitation event of a duration such that the entire watershed contributes simultaneously. Typically, this duration corresponds to the concentration time (*tc*), which is defined as the time taken by water to travel from the furthest point of the watershed to its outlet. Values of *tc* were calculated using the methodology presented by Kirpich [36] and the one proposed by Chow [37].

*tc*(*Kirpich*) = 0.000325(*L*/(*S* × 0.5))^0.77^ (2)

*tc*(*Chow*) = 0.00116(*L*/(*S* × 0.5))^0.64^ (3)

where *tc* is the concentration time (hours), *L* is the length of the main channel (m), and S its slope (m/m). For calculating the slope of the main channel (*S*), the graphical method of equivalent area was used, while the channel length (*L*) was measured using QGIS 3.22.11 software [34]. To determine the concentration time (*tc*), an average was computed between the two values obtained using the Kirpich (Equation (2)) and Chow (Equation (3)) methods. Peak flow rates for various return periods were calculated using the Rational Method [38].

*Qmax* = *C i A k* (4)

In the Rational Method (Equation (4)), *Qmax* is the value of the maximum water flow rate in m^3^/s expected at the considered position, *C* is an adimensional coefficient that takes into account the surface runoff fraction of rainfall, *i* is the maximum rainfall intensity expected in the selected area expressed in mm/h, *A* is the area in km^2^ of the catchment, and *k* is an adimensional constant used to correlate the units of measure of the other parameters. For determining the runoff coefficient (*C*), the values suggested by Benini [39] were adopted. This method is based on standardized tables that provide *C* values according to soil characteristics, slope angle, and land use. The study area was remotely divided into four sectors based on observations from satellite maps, with surface types categorized as meadow, debris, and forest. The percentage of each sector related to the total area (100%) was calculated. A weighted average of all land-use-specific runoff coefficients (*C*) was then computed to obtain the corrected runoff coefficient, which was used in the Rational Method (Equation (4)).

To determine the critical rainfall intensity (*ic*), the previously calculated concentration time (*tc*) was used. Critical intensities for various return periods were calculated as the ratio of rainfall depths of duration equal to the concentration time (*hc*)—obtained by substituting *tc* for *t* in the equation (Equation (1))—to the concentration time itself. The critical intensity is given by the formula:

*i_c_* = *h_c_*/*t_c_* (5)

The Rational Method (Equation (4)) is not the only approach available for calculating the flow rate. The Volumetric Method proposed by Armanini [40] allows for the estimation of the maximum flow rate of the surge by knowing the liquid discharge and its concentration, according to the following relationship:

(6) $$\mathrm{Qmax}=\mathrm{Ql}\;(\frac{c*}{c*-c})$$

where *Qmax* is the maximum flow rate of the debris flow (m^3^/s), *Ql* is the maximum liquid flow rate (m^3^/s), *c* is the debris concentration (dimensionless), and *c*∗ is the maximum packing concentration of solid material at rest (typically 0.65 ÷ 0.750.65, dimensionless).

On the other hand, according to Takahashi [41], for slopes exceeding 20°, the debris concentration (*c)* is equal to 0.9*c*∗.

*Qmax* = 10 *Ql* (7)

Once the peak flow rates were calculated using both the Rational Method (Equation (4)) and the Volumetric Method proposed by Takahashi (Equation (7)), the total water volume accumulated in the watershed was determined by multiplying the peak flow rates by concentration time.

By assuming the water volume to be 30% of the total flow volume, as suggested by Coussot and Meunier [42] and Hungr et al. [13], the remaining 70% of the volume of solid sediments transported was calculated. The total debris flow volume was obtained by summing the solid and liquid portions.

*Vol tot* = *Vol_aqua_*_(30%)_ + V*ol_solid_*_(70%)_ (8)

### 3.3. Grain Size Distribution

To determine the grain size distribution of the material, the dimensions of a significant number of rock samples from the debris flow deposits were measured. Some of them were measured directly in situ, while the largest number of measurements were performed remotely on digital images of the debris. Such images were scaled and processed with ImageJ 1.54K, a freely available Java-based public-domain image processing program developed at the National Institutes of Health (NIH) [43]. The analysis of the 40 in situ samples allowed the determination of proportional relationships among the three dimensions of the analyzed blocks. For the samples measured on images, only the two visible dimensions were recorded. The third dimension was approximated using the previously calculated ratios, averaged arithmetically from the in situ measurements.

Once the dimensions of all the samples were obtained, their volumes were calculated. Assuming the samples to be perfectly cubic—given their generally equidimensional appearance—the cube root of the volumes was calculated to obtain the diagonal values. These diagonal values were processed using Excel to derive absolute and relative frequency values for the grain size classes under investigation.

A grain size distribution curve (d-%passing) was constructed. With this information, it was possible to classify the flow that occurred at the study site. This was achieved by referencing the diagram developed by Coussot and Meunier [42]. The diagram classifies flow types based on the solid fraction of the event and the percentage of fine material (<0.04 mm), which, in this case, accounts for less than 10% of the total landslide volume.

### 3.4. The Debris Flow Numerical Model

To simulate the studied gravitational phenomena, the three-dimensional hydraulic modelling software HEC-RAS was used, where time is considered as the third dimension [44,45,46].

For a three-dimensional simulation with HEC-RAS, such as those conducted in this paper, the user must specify the morphology of the studied area, the terrain characteristics, and the inflow and outflow water discharges of the system. Additionally, the calculation method (steady flow, quasi-unsteady flow, or unsteady flow), the maximum number of iterations, the computation time, and the temporal resolution of the results must be defined. The geometric and morphological characteristics of the site are directly provided through a DTM.

To develop the models, it is necessary to import the DTM of the study area and to overlay it with a series of geometries that the software refers to when creating the simulation [27]. Specifically, the following are included:

- A model perimeter within which the simulation is carried out.
- Boundary condition lines: an inflow line located upstream of the study area and an outflow line located downstream. These define the start and end points of the model.
- Breaklines, one for each primary channel identified in the simulation perimeter. These lines are necessary to guide the software in determining the directions in which the flow will develop.
- Flow data, including the flood hydrograph.

### 3.5. Photogrammetric Survey and DTM

**Figure 7:**
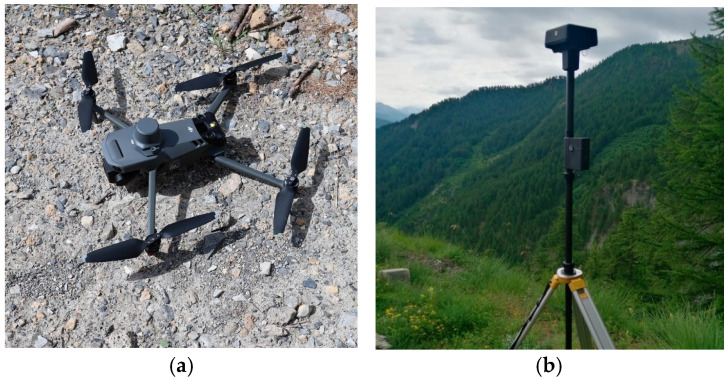
(**a**) DJI Mavic 3 Enterprise drone used for the photogrammetric survey; (**b**) RTK antenna.

The DTM provided by the Piedmont Region, with a spatial resolution of 5 × 5 m and a vertical accuracy of approximately 0.50 m, was considered insufficiently detailed for the numerical modeling in order to produce consistent and reliable results. Therefore, an unmanned aerial vehicle (UAV) survey was planned to be conducted. The goal was to produce a DTM with a 0.50 × 0.50 m spatial resolution. Specifically, a series of UAV flights were conducted using a DJI Mavic 3 Enterprise drone on 3 July 2024 (Figure 7a). The morphological condition of the area, namely the high slope and the presence of vegetation, did not consent to designing the flight route before accessing the site. The flights were carried out with a manual control within a visual line of sight, ensuring the highest overlap among images. An RTK antenna (D-RTK 2 by DJI: Shenzhen, China) was employed as a high-precision GNSS receiver integrated with the drone (Figure 7b). This antenna supports all major global satellite navigation systems and provides real-time differential corrections, achieving centimeter-level positioning accuracy of image shooting positions [47,48].

During the flights, the drone captured photographs using both a nadir position of the camera (approximately vertical with respect to the UAV), a frontal position (approximately horizontal with respect to the UAV), and a position approximately angled by 45°. This methodology enables an accurate representation of both vertical and horizontal features. To ensure optimal survey accuracy, an overlap of at least 80% between consecutive images along a flight line and 60% between adjacent flight lines was maintained. The UAV flew about 50 m above the surface, following its variable slope. A total of four flights were conducted across the entire study area, resulting in over 700 images (resolution 5472 × 3648 pixels). These images were divided into sequences, each covering specific portions of the studied area. The image sequences were processed using the photogrammetric software Zephyr (3Dflow) [49]. The software, based on Structure from Motion algorithms [50], processed the sequences of images captured from multiple viewpoints to reconstruct the spatial coordinates of identifiable points contained in the images. This process generated a georeferenced, scaled point cloud representing the surface of the study area, with an average point density of 100 pts/m^2^. Resulting point clouds were imported into the software CloudCompare 2.13.2 [51] and merged to reconstruct the entire study area. Finally, a 2D DTM grid raster file was generated using the same software. This raster file was employed as the cartographic base in ArcMap 10.8.2 software (ESRI) for the realization of the geomorphological map of the area and as input for debris flow modeling.

## 4. Results

### 4.1. Geomorphological Survey and Mapping of the Rio Inferno Watershed

**Figure 8:**
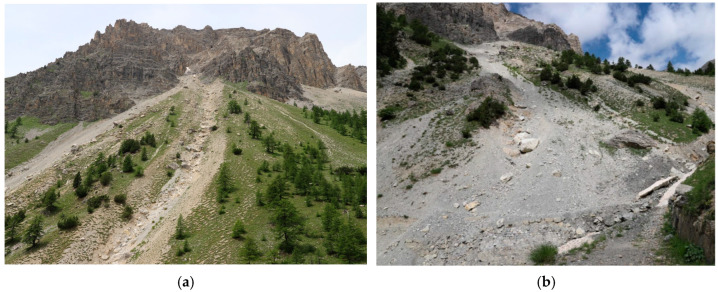
(**a**) Steep inclination of the rocky carbonatic walls; (**b**) debris accumulations at their foot.

The area shows morphologies that represent the expression of both recent and past geomorphological processes. For each morphogenetic agent, both erosional and depositional landforms have been observed. Morphologies and deposits related to gravity dominate much of the studied area, making gravity the primary morphogenetic agent and the main cause of instability in the site. In the entire western sector of the area, extensive scarps can be observed (Figure 8a) as the result of the contrast between exposed vertical rock walls and the deposits at their foot (Figure 8b), which have formed due to gravitational processes.

Based on the position of the debris relative to the slope and its disposition, it is possible to distinguish three main types of depositional morphology associated with this morphogenetic agent: detrital fans, debris cones, and debris-flow cones, along with their respective gravitational runoff channels. By observing the degree of revegetation of the debris deposits, it is possible to determine whether the gravitational process that generated them is active or inactive. It is also possible to identify in the site morphologies related to snow, surface water runoff processes, and past glacial activity as moraines and glacial detrital located on the north side of the area. Colluvial activity reshapes the glacial and gravitational deposits, creating areas where it is impossible to distinguish a single agent responsible for their genesis. These deposits are referred to as mixed-origin deposits. They tend to be more vegetated compared to other recognized deposits.

**Figure 9:**
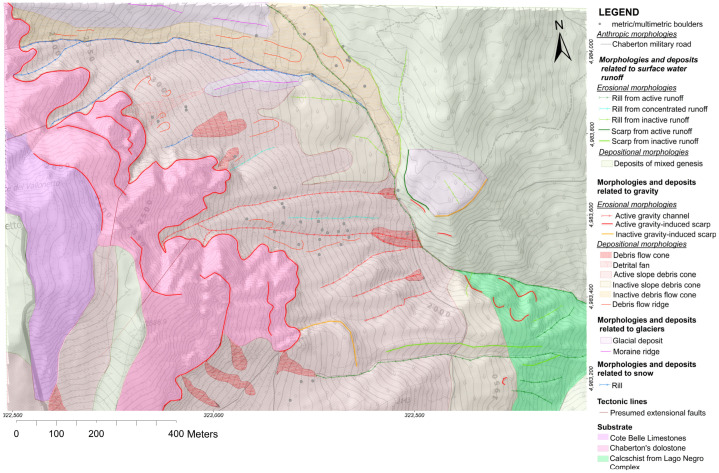
The geomorphological map of the area specifically created for this study. Coordinates in WGS 84 UTM zone 32N. The cartographic elements in red and orange represent landforms shaped by gravity: red indicates active forms, while orange denotes inactive ones. Purple is used for glacial landforms, while blue represents nivation-related features. Landforms shaped by surface water processes are depicted in green. In the legend, landforms are further classified as depositional or erosional.

The geomorphological study conducted in the area led to the creation of the geomorphological map shown in Figure 9.

### 4.2. Computation of Input Parameters

**Figure 10:**
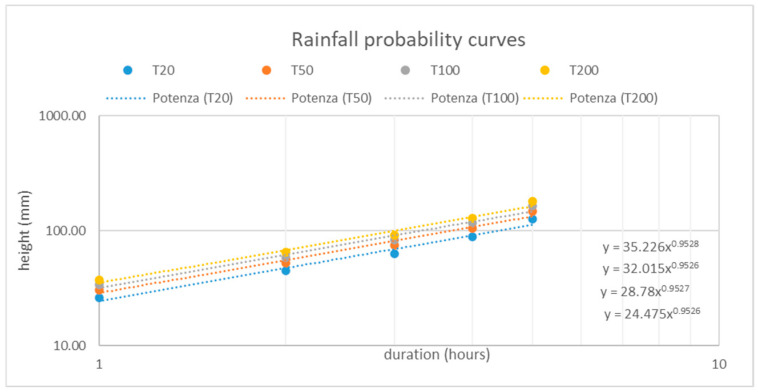
Rainfall probability curves for the four return periods considered (20, 50, 100, and 200 years), plotted using the Gumbel method (Equation (1)) on a logarithmic scale.

Based on data from the ARPA Piemonte portal [28] concerning rainfall durations of 1, 3, 6, 12, and 24 h in the watershed for the 25 considered cells and the four return periods, all rainfall values (in mm) were averaged. Using these averages, rainfall probability curves were constructed for all four return periods (Figure 10). The data points, plotted on a logarithmic diagram, were interpolated using an exponential trendline, enabling the derivation of the parameters *a* and *n* from the interpolation curve equation (Equation (1)), which appears linear in the logarithmic diagram.

**Figure 11:**
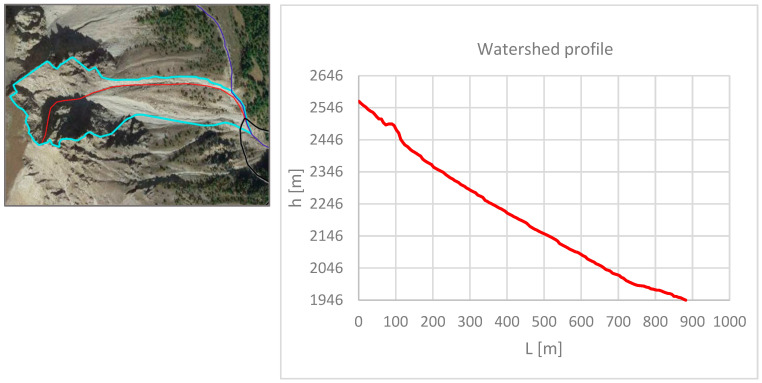
Profile of a reference section (highlighted in red in the side panel) height-length for the watershed. The light blue line indicates the extension of the watershed.

To quantify the concentration time (tc), as outlined in Section 2 and Section 3, the morphometric parameters of the watershed were determined. The watershed’s length (L) and slope (S) were calculated by creating a reference section using QGIS software 3.22.11 (Figure 11), which was subsequently imported into Excel for deriving the required parameters (Table 1).

The more conservative approach was to adopt the shorter concentration time calculated using Kirpich’s formula (tc = 4 min, 12 s), as the result from Chow’s method is approximately two minutes longer. The decision to avoid averaging the two times was based on the principle that shorter times correspond to more concentrated flows over a shorter interval, leading to more intense flow rate peaks. Under such conditions, downstream hydraulic systems have less time to respond, increasing the risk of overload and flooding. Adopting shorter times therefore aligns with the most precautionary conditions.

With the known tc, the critical rainfall depth (hc) and intensity (ic) were calculated.

**Figure 12:**
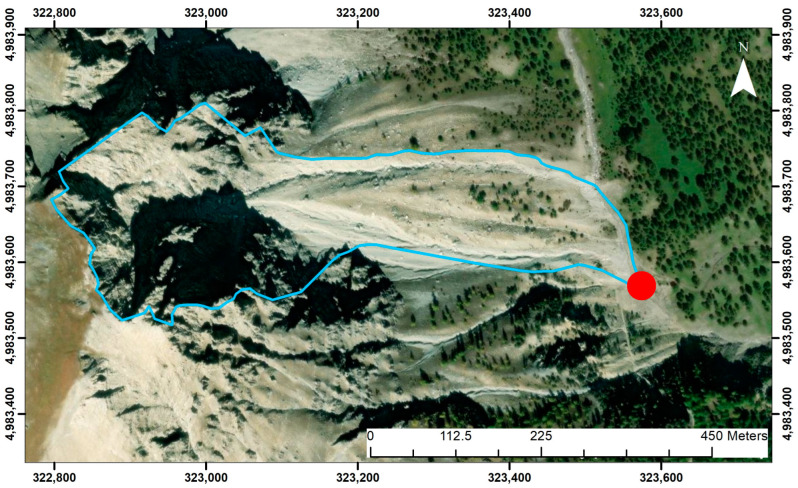
Delineation of the investigated watershed. The closure point of the watershed (red dot) is located at the confluence with the Rio Inferno. Coordinates in WGS 84 UTM zone 32N.

Considering the confluence point between the debris flow channel and the surface runoff channel (marked in Figure 12) as the watershed closure, the watershed geometry was drawn in QGIS [34].

**Figure 13:**
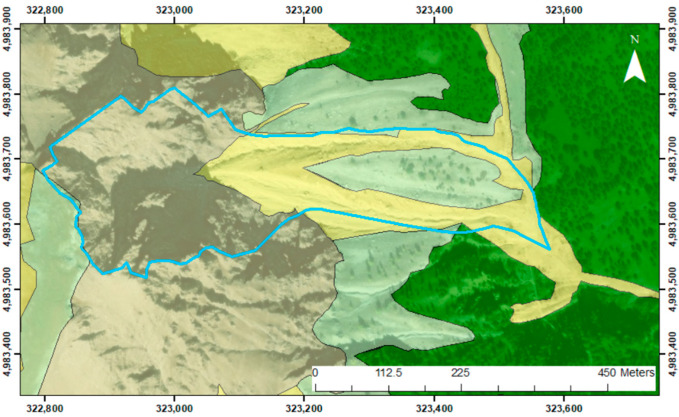
Subdivision of the studied area into macrodomains: forest (dark green), meadow (light green), debris (yellow), and rock (grey) to determine the C coefficient for the Rational Method’s Formula (4). Coordinates in WGS 84 UTM zone 32N.

To determine the C coefficient (Equation (4)), the watershed of the study area was remotely divided into four sectors (Figure 13).

For the Rational Method (Equation (4)), the required parameters were estimated as follows. K = 0.278 K. The total runoff coefficient was obtained through a weighted average of infiltration coefficients for each domain presented in Figure 12, Ctot = 0.87. The watershed area (A) was measured using topographic data from the regional DTM via QGIS software and is equal to 0.125 km^2^.

Using these parameters, flow rates (Table 2) and volumes (Table 3) corresponding to each of the four return periods were calculated. The debris flow peak flow rate using the Rational Method (MR) was obtained considering a solid concentration of 30% in the water-debris mixture, as recommended by Coussot and Meunier [42] for alpine environments.

### 4.3. Construction of the Grain Size Distribution

**Figure 14:**
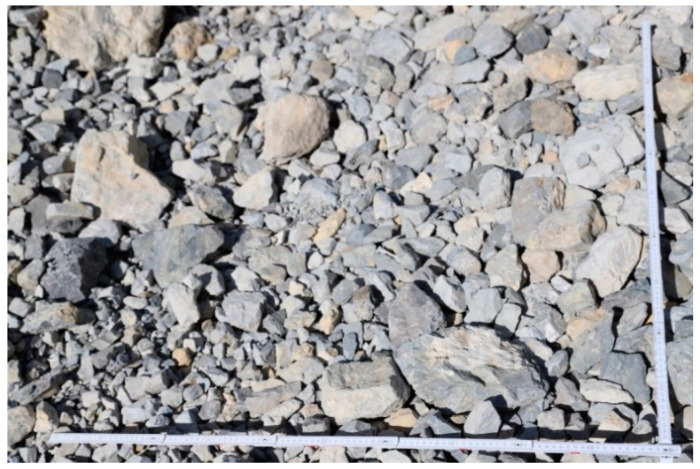
Example of an image used for the measurements of rock blocks with ImageJ software [43].

To determine the grain size distribution of the material, the dimensions of 227 rock samples from the debris flow deposits were first measured. The three dimensions of 40 samples were measured directly in situ, while the remaining 187 samples’ measurements were performed remotely, through ImageJ software [43], on 9 images containing two perpendicular scale bars (Figure 14). The analysis of the 40 in situ samples allowed the determination of this specific relation among the three dimensions of the analyzed blocks: 1:1:0.8. The in situ measurements confirmed the almost equidimensional shape of the debris. For the 187 samples measured on images, only the two visible dimensions were recorded; the third dimension was approximated using the previously calculated ratios.

**Figure 15:**
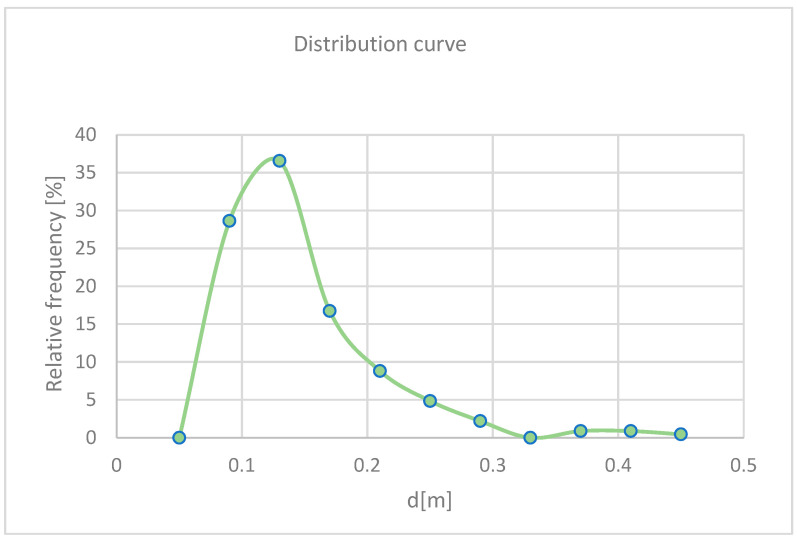
Relative frequency of particle size of the analyzed samples: the maximum frequency corresponds to 0.13 m.

To construct the grain size distribution curve characteristic of the debris flow deposits in the study site, the minimum diagonal value, namely the minimum size of a particle, was set to 0.05 m. This choice was made coherently with the millimetric graduation of the scale bar, used as reference to perform the measurements. The obtained relative frequency curve is shown in Figure 15.

**Figure 16:**
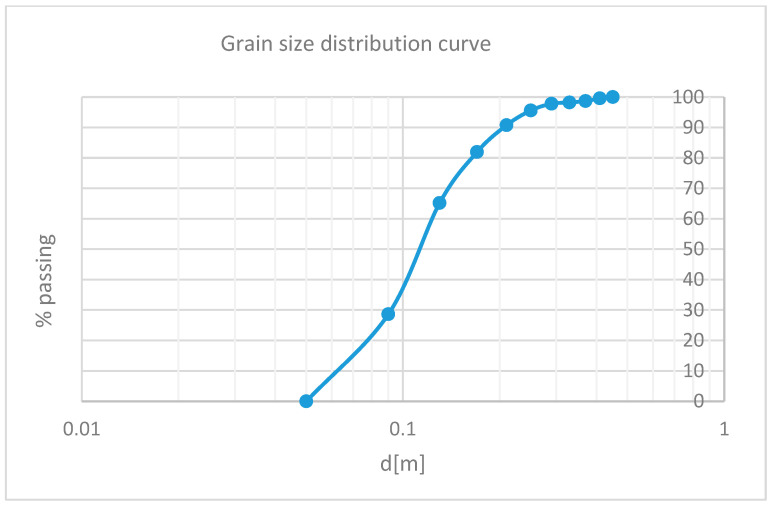
Grain size distribution curve obtained.

As observed, the relative frequency curve has a left tail constrained by the minimum imposed value of 0.05 m (theoretically, the physical limit corresponds to zero), while the right tail is limited by the dimensions of the photographed area used for measurements (approximately 1 m × 1 m). By calculating the absolute frequency for the considered classes, the grain size distribution curve was obtained (Figure 16).

Considering the characteristics of the debris observed in the field, the trend of the obtained grain size distribution curve, and the classification criteria proposed by Coussot and Meunier [42] based on the granulometric distribution curve of debris flow deposits, the event can be classified as a granular debris flow, in which the fine fraction accounts for less than 10% of the total landslide volume.

### 4.4. Set up of the Debris Flow Numerical Model

**Figure 17:**
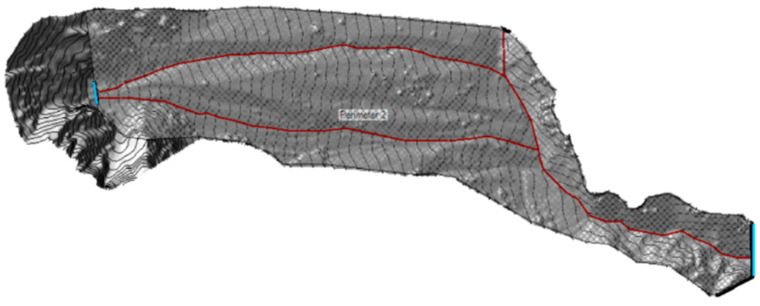
The image shows a screenshot of the RAS Mapper from HEC-RAS, with the inflow boundary condition line (left) and outflow boundary condition line (right) highlighted in blue. The red lines represent the breaklines corresponding to the main channels. The grey area represents the simulation area.

**Figure 18:**
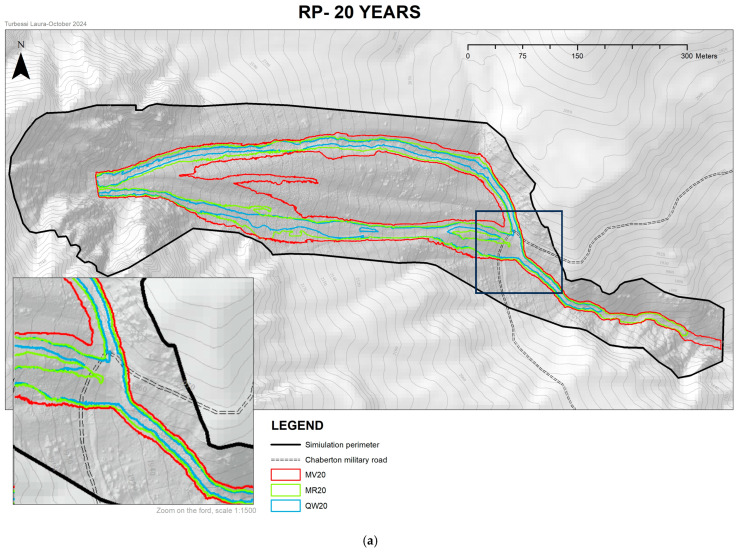

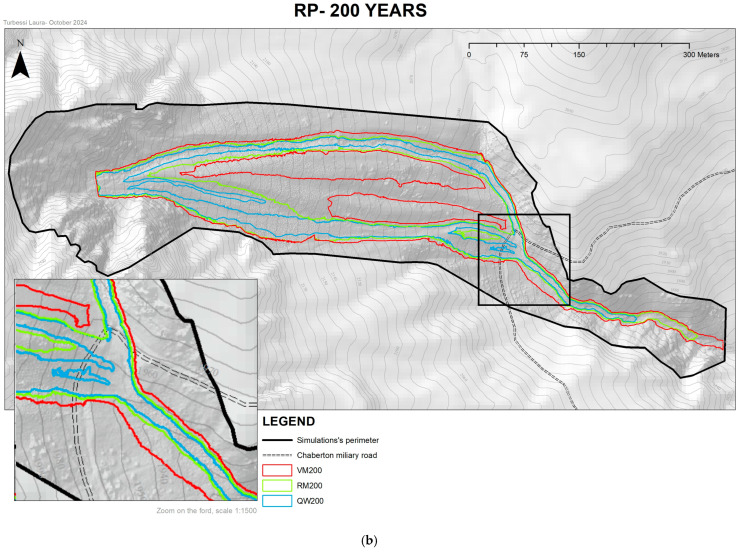
Maps of the results of the HEC-RAS simulations obtained considering discharges calculated with the Volumetric Method (VM-red) (7), Rational Method (RM-green) (4), and Rational Method considering only the water discharges (QW-blue) (4) for return periods of (**a**) 20 years and (**b**) 200 years.

The unsteady-flow model was adopted to carry out the simulations in this study. This model is based on three main input parameters: a sufficiently detailed DTM, debris flow volumes, and the corresponding flood hydrographs. Due to the inability to determine the characteristics of the finer granulometric fraction of the deposit and the final position reached by the debris flow cone, caused by its dispersion in the valley floor, it was necessary to use models requiring only input parameters that could be derived or estimated. Consequently, for this specific case, the landslide event was simulated by considering the fluid as Newtonian. This approach allows the creation of flow models based on the available data while adopting a conservative perspective. Specifically, this assumption results in the highest possible velocities and flow distances, as it does not consider the internal friction generated by the solid fraction of the flow. For the model development in HEC-RAS, it was necessary to delineate, in addition to the simulation perimeter, two boundary condition lines and three breaklines, one for each of the main channels identified within the simulation perimeter as shown in Figure 17. The constructed model covers the entire watershed and a portion of the area downstream of the ford of the Chaberton Military Road (Figure 18).

A total of twelve models were developed. Each model features distinct hydrographs corresponding to the calculated outflow for water alone (QW), the debris flow according to the Rational Method (RM), and the Takahashi Volumetric Method (VM) for the four different return periods. The flow hydrographs assigned to the twelve models all feature an initial and final discharge of zero, with a peak flow rate corresponding to the calculated concentration time. HEC-RAS requires hydrographs to be imported in tabular format. A Data Time Interval—the time elapsed between consecutive flow values—of 10 s and a total simulation duration of 8 min was set. By entering the initial, peak, and final flow rate values, the software automatically interpolates the missing values and generates the hydrograph. Once the flow hydrograph was imported, a Computation Interval of 10 s (consistent with the previously set Data Time Interval) and a Hydrograph, Detailed, and Mapping Output Interval of 1 min were configured. Following this setup, the simulation was executed. After computation, the results plotted in the RAS Mapper were examined, focusing exclusively on the models related to the flow height profiles. These results were exported both as shapefiles and raster data to be processed in GIS. The models generated by HEC-RAS were then digitally processed in ArcMap to produce four maps, each corresponding to one of the considered return periods. These maps illustrate the maximum extent of the simulated flows and allow the visualization of the potential impact of debris flows on the Chaberton Military Road near the Rio Inferno ford as a function of the return period. The HEC-RAS simulations produced flow models with maximum velocities reaching 15 m/s in the central sections of channels located in the steeper portions of the slope.

The most conservative models, based on flow rates calculated using the Takahashi Volumetric Method (VM), simulate phenomena with maximum flow heights above the topographic surface ranging between 11 m (TR = 20 years) and 13 m (TR = 200 years). Models developed using discharges calculated with the Rational Method (RM) produced maximum heights between 6.5 m and 8 m for return periods of 20 and 200 years, respectively. As expected, flows modeled using the Rational Method for water alone (QW) simulated smaller extents than the previous models, with flow heights not exceeding 5.5 m, 

## 5. Discussion

The combination of the actions of numerous morphogenetic agents that have affected the study area over time has led to the formation of a landscape that today remains highly active. The Rio Inferno watershed represents a region highly susceptible to hydrogeological instability due to the persistent gravitational phenomena and active runoff processes. The evolution of the direct fault systems (Section 2) fractures the dolomitic vertical walls that are hundreds of meters high and located throughout the western sector of the study area (shown in Figure 7a,b). Their deterioration forms a continuous source of detrital material that tends to accumulate in correspondence with the slope changes at the base of these rock walls. Due to the combined effect of spring snowmelt and intense summer rainfall, the water mobilizes the material deposited during the colder and drier seasons, generating debris flows intense enough to compromise the integrity of the Chaberton Military Road.

This study conducted on the granulometric distribution of debris allows the classification of the observed debris flow deposits as generated by granular debris flows. The granulometric curve obtained is steep, indicating a homogeneous distribution of clast sizes. Specifically, 65% of the analyzed clasts range between 0.09 and 0.13 m in size, while 82% fall within the range of 0.09 to 0.17 m. Considering that the debris flows originate from detritus coming from the dolomitic walls, it is reasonable to assume that the clast sizes are consistent with the fracture spacing in the walls from which the material detached. This holds true except for fragmentation processes that occurred after detachment.

Considering that debris flows at the latitude and altitude of the Fenils sector are typically composed of 70% granular solid [42], it was possible to estimate the total volume of the most critical debris flows. The involved volumes range from 2526 m^3^ to 3635 m^3^ (Table 3), depending on the return period considered. Although, according to Fell’s classification [52], landslides involving volumes between 500 and 5000 m^3^ are considered very small-scale, low-intensity phenomena, the hazard posed by debris flows is not based on their size but on the velocity they can achieve. On the other hand, according to the classification by Hungr [53], later refined and rationalized by Cruden and Varnes [11], a debris flow with characteristics like those in the Rio Inferno watershed and average velocities of 10–15 m/s would be classified as extremely rapid and belonging to the highest hazard category. Considering that the study area is poorly anthropized, with the only identifiable human element being the Chaberton Military Road—non-drivable and primarily used by hikers—it is unlikely that such events could cause significant damage to infrastructure or loss of life. However, the recent destruction of the Rio Inferno ford and the complete burial of the trail section crossing the studied watershed highlight the need for effective interventions to restore the road and reopen the trail connecting Fenils to the Chaberton Fort.

The development of debris flow models in the HEC-RAS environment serves a dual purpose: to visualize the road segments affected by critical debris flows for various return periods and to provide parameters such as velocity and flow height for modeled scenarios. The HEC-RAS simulations showed flow models with maximum velocities of 15 m/s at the center of channels located in the steepest sections of the slope. The velocity of the debris flows is not strictly dependent on the return period considered. For both methods used to calculate flow rates, the maximum velocities reach 15 m/s across all return periods. This is due to the high steepness of the slope, which enables the flows to achieve very high velocities even under less intense precipitation events.

The extent reached by the debris flows confirms that the flows affect the exact section of the trail currently buried by landslide debris. This demonstrates the consistency of the models with real-world conditions and validates the reliability of the simulations. The segment of the road affected by the flows varies by approximately 10 m depending on the discharge calculation method used. Models based on discharge values derived from the Takahashi Volumetric Method (Equation (7)) show a road segment affected by flows of approximately 76 m for a 200-year return period and 67.08 m for a 20-year return period. Similarly, models using discharge values from the Rational Method (Equation (4)) indicate an affected segment of 66.72 m for a 200-year return period and 59.91 m for a 20-year return period.

For the restoration and reopening of the Chaberton Military Road, at least for pedestrian use by hikers, the study of the Rio Inferno watershed highlights the need to account for the frequent recurrence of debris flow phenomena in the area. The cheapest and simplest intervention could involve installing a series of fords to allow debris flows to pass downstream, combined with localized road reinforcements and continuous maintenance to clear the trail of detrital material. A more complex and significantly more expensive structural intervention would involve constructing a suspension bridge approximately 200 m long, bypassing the section of the trail most likely to be affected by debris flows.

## 6. Conclusions

In extreme alpine environments that are not easily accessible, the remotely performed geomorphological mapping and the analysis of the main active geomorphological processes are essential for understanding the source areas and the characteristics of rapid landslide phenomena. Such knowledge should be a reliable tool to plan how to mitigate landslide risk.

The study of the Rio Inferno watershed has highlighted the intense activity of morphogenetic agents, such as water and gravity. These phenomena cover 40% of the watershed and require detailed characterization to plan and design interventions to restore the viability of the Chaberton Military Road. The UAV photogrammetric survey is of fundamental importance for the reconstruction of high-resolution DTMs, which provide a basis for both remote geomorphological surveying and the modeling of gravitational phenomena and flows.

The use of models that treat the flow as a Newtonian fluid provides profoundly conservative results, which assume the extreme condition of complete absence of internal friction in the solid fraction of the debris flow. These conservative outcomes serve as a basis for assessing the feasibility of potential future interventions in the area. The characterization and prediction of these phenomena are essential for designing engineering interventions to ensure road safety. This approach considers the almost certain recurrence of similar debris flow events with intensities comparable to those observed in the past, preventing damage to infrastructure and avoiding costly future interventions.

## Figures and Tables

**Table 1:** Watershed length, slope, and the concentration times calculated using both the Kirpich (Equation (2)) and Chow (Equation (3)) methods.

L [m]	881.77
S (Δh/L)	0.70
tc Kirpich—Equation (2) [min]	4.14
tc Chow—Equation (3) [min]	5.98

**Table 2:** Flow rates calculated using the Rational Method and the Takahashi Volumetric Method.

RP (Years)	QW (m^3^/s) [Equation (4)]	Qtot RM (m^3^/s) [Equation (4)]	Qtot VM (m^3^/s) [Equation (7)]
20	3.01	10.03	30.08
50	3.54	11.79	35.36
100	3.93	13.11	39.33
200	4.33	14.43	43.28

**Table 3:** Flood and debris flow volumes calculated according to the equation 8.

Vw (m^3^)	Vsolid (m^3^)	Vflow (m^3^)	Conc.
757.93	1768.50	2526.42	70%
891.00	2078.99	2969.98	70%
991.12	2312.62	3303.74	70%
1090.55	2544.62	3635.17	70%

## Data Availability

The raw data supporting the conclusions of this article will be made available by the authors on request.
